# Formulae for Insect Wingbeat Frequency

**DOI:** 10.1673/031.010.9601

**Published:** 2010-07-08

**Authors:** Michael A B Deakin

**Affiliations:** School of Mathematical Sciences, Monash University, Clayton, Vic, Australia

**Keywords:** dimensional analysis, insect flight, allometry

## Abstract

A formula is developed connecting the wingbeat frequency of insects with their masses and wing areas. It is derived first theoretically, using dimensional analysis, and then it is compared with published measurements. The formula discovered involves two parameters which dimensional analysis alone cannot determine. One of these is evaluated using one among many proposed semi-empirical relationships (the only one that stands scrutiny); the other by fitting a published dataset. It is found that the resulting equation, applying to insects in general, accords well with observation, and indeed is very close to being optimal (in a sense to be defined).

## Introduction

The question of determining the wingbeat frequency of an insect has been the subject of many studies. Much of this is now readily available in a recent book (Dudley 2000), but there are also useful accounts by Azuma (2006) and Weis-Fogh (1977); another work (Greenewalt 1962) has achieved the status of a classic, and a more recent compendium of results, (Byrne et al. 1988) provides a lot of material in accessible and useful form. This last provides the basic data to be analyzed here.

The object of this study was to develop a formula for the wingbeat frequency *n* of an insect in terms of its mass *m* and its wing area *A*. The method used was dimensional analysis, which proceeds from a bare minimum of assumptions. In particular, it avoids the production of a detailed model of the process being analyzed; rather, it places necessary restrictions on more specific models that can be developed later. The basic assumptions are that a formula of the type sought actually exists, and that this formula should be independent of the units of measurement involved.

The formula to be derived may be classified as a “double allometry” in that the wingbeat frequency is given as a product of powers of mass and wing-area. This should be contrasted with the (single) allometric formulae advanced by earlier authors.

Several authors (e.g. *inter alia* Chadwick 1953, Rashevsky 1960, Greenewalt 1962, Crawford 1971, Dudley 2000) have developed or promulgated suggested formulae for *n*. These authors used various suggested physical characteristics of the insects under study. Many simply sought (single) allometries between *n* and some other variable, such as *m* or a length-scale *l*. The present author (Deakin 1970) proposed a new formula, and part of the purpose of the present paper is to show that it still remains a good one, in some important ways the best. Whereas the earlier paper developed much of the theory, it lacked an extensive and reliable dataset against which it could be checked. That deficiency is addressed in the present analysis.

Furthermore it is found that, of all the possible doubly allometric formulae giving *n* in terms of *m* and *A*, this one is optimal in the sense of minimizing the total least square error.

In addition to the variables *n, m* and *A*, three physical parameters are also discussed. These three parameters, although essentially constant, are nonetheless relevant to the process of flight and so must be considered as (at least potentially) occurring in the formula sought. These are: 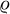, the density of the air; *µ*, the viscosity of air; and *g*, the acceleration due to gravity.

The most controversial aspect (indeed really the *only* controversial aspect) of dimensional analysis lies in the initial choice of variables and parameters. In the present case there is little difficulty with the choice of parameters. However there has been some disagreement over the variables. That *n* and *m* should be involved is almost axiomatic, but the use of *A* is not so generally adopted. Greenewalt (1962) lists various other variables that have been measured, either with a view to developing (simple) allometric equations or else in pursuit of some mechanical model or other. The most plausible of these other variables is wing-length, but others have also been tried (e.g. the moment of inertia of the wings, whose accurate measurement is surely susceptible of great experimental difficulty). However, as flight is dependent on the provision of a lift-force, it seems most natural to concentrate on the area of the lifting surface: the wings. Moreover wing-area is readily measured. Thus, if a single variable is to be selected from the various candidates, then wing-area is a front-runner. This point is implicit in the work of Byrne et al. (1988), who chose to list this quantity rather than any of the others that have been proposed.

In the present context, the use of SI units is clumsy, as insects are too small to have their masses measured in kilograms and their wing areas measured in square meters. Instead the older cgs system in which masses are given in grams and wing areas in square centimeters was used. This is the convention adopted by Byrne et al. (1988), who provided the relevant values for *m, A*, and *n* in these units for a list of over 150 insect species. This is the dataset to be used here.

The values of *m* in this dataset range from 3.3×10^-5^ gm (for a small whitefly, *Bemisia tabaci;* Homoptera: Aleyrodidae) to 2.809 gm (for a large moth, *Oryba achemenides*; Lepidoptera: Sphingidae). The values of *A* range from 0.0096 square cm (for another small whitefly, *Trialeurodes abutilonea;* Homoptera: Aleyrodidae) to 120 square cm (for another large moth, the Great Peacock Moth, *Saturnia pyri*; Lepidoptera: Saturniidae). The values of *n* range from 6 hertz (for a gracile butterfly, *Pieris napi*; Lepidoptera: Pieridae) to 480 hertz (for the yellow-fever mosquito, *Aedes aegypti;* Diptera: Culcidae).

In cgs units, the values of the parameters involved are: α = 1.2 × 10^-3^ gm/cc, µ = 1.8 × 10^-4^ poise, and g = 980 cm/sec/sec.

### Dimensional Analysis

Dimensional analysis proceeds by listing the variables and parameters involved in a problem and then looking at the types of measurements they involve. In the present context, there are three basic units (mass M, length L and time T) that come into consideration. All the other quantities (variables and parameters, six in all) to be discussed are measured in terms of units derived from the three basic units and expressible as products of powers of them. These powers give the *dimensions* of the quantities.

The variables and parameters together with their dimensions are shown below

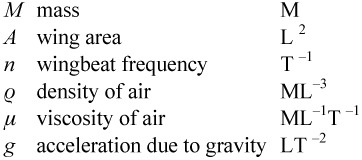

If the formula sought is to be valid in all systems of units, then it must be expressible in terms of quantities that are free of units, so-called *dimensionless ratios*. In the present context, the six quantities listed above involve the three basic units specified above. Because of a result known as the Buckingham *π*-theorem (see, e.g. Barenblatt 1987), we expect three (3 = 6 – 3) independent dimensionless ratios to arise from this situation.

These three ratios may be constructed in many ways, all mathematically equivalent but some more useful than others. The following choice is convenient and moreover relates to specialist labels given to each of the ratios and named below:

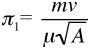

(essentially a Reynolds Number),

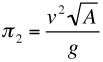

(essentially the square of a Froude number),

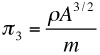

(a buoyancy factor)

The lowest value of the Reynolds number *π*_1_ is 8.47 (for a small aphid, *Aphis gossypii;* Hemiptera: Aphididae), but apart from a few species of aphids and whiteflies, values in excess of 100 and more typically of the order of 1000 are the rule. Large Reynolds numbers imply that viscosity is unimportant. The low values of *π*_2_ applying in the exceptional cases may underlie the observation of Byrne et al. (1988) that such insects manifest different patterns from those applying to larger ones and their suggestion that their mechanisms of flight may be different. Thus, apart perhaps from these few possible exceptions, the neglect of viscosity is justified.

The values of *π*_2_ vary between 0.02 (for *P. napi*) and 45 (for *Ae. aegypti*), with values between 1 and 10 being the usual case.

The buoyancy factor varies between 0.003 (for *Pieris brassicae;* Lepidoptera: Pieridae) and 1.58 (for *A. gossypii*). Typically, this ratio is “small” with a mean value a little below 0.1.

As we are neglecting the Reynolds Number *π*_1_; the formula we seek is of the form *F* (*π*_2_, *π*_3_) = 0 which may be rewritten as

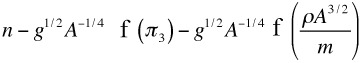

f(.) is an unknown function. Suppose however that f(*π*_3_) may be expanded by means of a Frobenius series (a very general functional form):

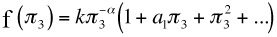

where *k*, *α* are dimensionless constants, i.e. pure numbers.

The leading power is written as negative for later convenience. Because *π*_3_ is small, neglect all terms beyond the leading one, and so find

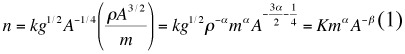

where *K (=kg^1/2^ ρ-α)* is a constant to be determined (but not a dimensionless one), and

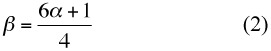

Equation (1) has the form described above as a “double allometry”.

### Further analysis

Equation (1), supplemented by (2), is as far as dimensional analysis alone can take us. The further determination of *K* and *α* must depend either on experimental evidence or else on some more sophisticated analysis.

**Figure 1.**
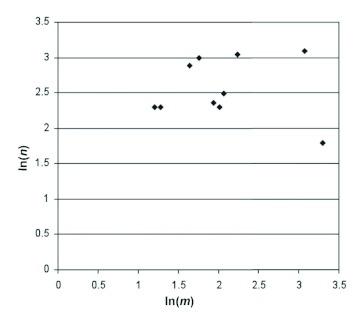
A plot of ln(*v*)versus —ln (*m*) for butterflies. If any of the proposed allometric laws applied to butterflies, these points would exhibit significant positive correlation, but they do not. High quality figures are available online.

Many of the proposed empirical laws use some (simple) allometric equation or other to provide data for further analysis. Greenewalt (1962) in particular considered all insects (and also bats and birds other than humming-birds) to be approximately geometrically similar, although he recognized the difficulty of pushing such analysis too far. For insects, he proposed (his Figure 12) the relation n *nαl^-1.15^* where *l* is the wing-length. His Figure 1 thus allows the deduction *nαm*^-0.383^ because he has *m* scaling as *l^3^* similarity theory would predict.

However, other such relations have also been posited. Rashevsky (1960) posited on theoretical grounds that for “approximately similar” insects *nαm^-1^* and found some support for this view in the literature. Weis-Fogh (1977, p. 416) has *nαl*^-1^ and thus *nαm*^-1/3^Dudley (2000, figure 3.3B) derives a relation *nαm*^-0.24^. It is clear that there is no agreement on this matter. Neither Greenewalt nor Dudley are very convincing. Greenewalt (1962) offers a graph, which only achieves its result after the insects have been divided (rather arbitrarily) into four distinct groups. Dudley (2000), in an incompletely described graph, includes humming birds along with insects and achieves a value *r*^2^ = 0.17, i.e. a correlation coefficient of 0.41 for a line of best fit through a very scattered cloud of points. Rashevsky (1960) considered only a small dataset and also misdescribed the relevant graph. Weis-Fogh (1977) offers a theoretical analysis for his “general interspecific rule,” but does not test it against field data. The same law is attributed by Dudley (2000, p. 90) to Hill (1950), but Hill does not discuss insects.

Any attempt to derive a formula on the basis of geometrical similarity encounters the difficulty of deciding quite when insects are “approximately similar.” Clearly one cannot regard *all* insects as “approximately similar”, i.e. approximate scale models of one another. Diptera, for example, have quite different shapes from Lepidoptera. On the other hand, if any such formula is to be useful, it must not be so restricted in scope as to find no application at all. Here it is proposed as a test that if any such formula is to be meaningful, then it should apply to butterflies (Nymphalidae, Papilionidae, and Pieridae), and that we should find an approximate proportionality *n* 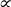 *m*^-γ^ for butterflies, where *γ* is a constant. That is to say that there should be significant positive correlation between the values of ln (*n*) and — ln (*m*) Instead, however, the result is as depicted in Figure 1. The correlation coefficient is -0.053 which (as well as being negative) is not significant.

Thus the attempt to find and use simple allometry between *n* and *m* will not provide the value of α.

### Other proposed empirical laws

Other laws have been advanced. Deakin (1970) considered two relationships proposed by Rashevsky (1960). The first of these has just already been discussed and dismissed (as it was, on rather less secure grounds, in that earlier paper). Rashevsky's other suggestion was that for insects of the same mass *n* 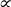 *m/A* the wingload, *L*.

If this is adjoined to the analysis, we find that Equation (1) becomes

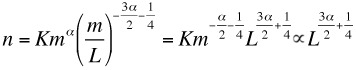

in the event that *m* is constant. If the index in this final term is to be 1, as the Rashevsky had it, then, as was shown in the earlier study, it follows at once that *α* = ½.

As a test of validity, we thus search the dataset for subsets of insects with approximately the same mass. This examination reveals:

1.A group of 8 species with masses around 0.025 gm
2.A group of 7 species with masses around 0.07 gm
3.A group of 10 species with masses around 0.1 gm
4.A group of 10 species with masses around 0.55 gm.

However, analysis of these sets shows only partial support for the effect claimed. If we look at the actual (observed) values of *n*, and compare these with those predicted by the empirical “law,” we find coefficients of correlation as set out below.


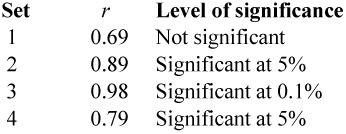


This provides *some* evidence that the relationship holds, but it is not as strong as one might like. However if we accept this “law” and its consequence *α* = ½, then the formula sought becomes

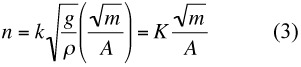

where *k* is a dimensionless constant, and thus *K* is also a constant, although not a dimensionless one. This was the result of the earlier analysis (Deakin 1970). A derivation of Equation (3) was later supplied by Crawford (1971), who employed a simple physical model. This is the double allometry mentioned above; it has the values *α* = 0.5, *β* = 1.

### Test of Equation (3)

**Figure 2.**
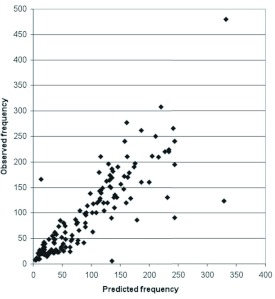
Predicted and observed values of *v*. High quality figures are available online.

If we test Equation (3) against the data from Byrne et al. (1988), we find the situation depicted in Figure 2, where the predicted values of the wingbeat frequency are given by means of the formula

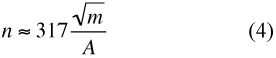

and the correlation coefficient *r* between this and the observed reality is 0.866, a highly significant figure (p < 0.001), and corresponding to r^2^ = 0.75 so that Equation (4) explains 75% of the variance in *n*.

### Fitting Equation (1)

Although Equation (3) provides good fit, it is possible to examine Equation (1) independently of the hypothesis that led to the specialization (3). A best fit of the form ln*n* = ln*K* + α ln*m* — β ln*A* to the data yields the relation

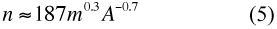

(i.e. *α* = 0.3, *β* = 0.7). As the theory predicts, Equation (2) is satisfied but the value of *α* is not what Equation (3) gives. The use of logarithmic variables in testing allometric relationships is widely used because it reduces the problem of finding a nonlinear regression to the computationally simpler problem of a linear one.

However, a best fit in the logarithmic sense is not necessarily a best fit in the original variables. A device adopted for computational convenience can actually produce incorrect answers. This effect is exemplified here. The values *α* = 0.3, *β* = 0.7, *K* = 187 produce a total squared error of 308,083.5 whereas the choice *α* = 0.5, *β* = 1, *K* = 317 gives 257,905.6. Indeed a computer search in the region of (*α, β, K*)-parameter space close to these values shows that this choice is almost optimal in the sense of minimizing the total squared error in *n*. (The best result in this sum of squared errors sense is given by the values *α* = 0.5, *β* = 0.98, *K* = 325, which does not represent a significant difference.) Equation (4) is thus not only better than Equation (5) but results in the best simple fit that can be achieved for an equation of the double allometric type.

## Discussion

The application of dimensional analysis to the problem of insect wingbeat frequency succeeds, producing good agreement with observation. However, the “empirical laws” formerly used to supplement it do not stand scrutiny so well. It might be said that the resulting equation is better than its derivation.

Nevertheless Equation (4) provides as good an agreement as can be hoped from a simple formula applied to so complex a problem. It explains 75% of the variance in *n*, whereas other claimed fits do not do nearly so well. Compare, for example, Dudley's (2000) value of 17%.

An interesting corollary follows from such studies. Comparing (e.g.) Pieridae with Apidae, we note the relatively gracile bodies and larger wings of the former and the relatively small wings and large masses of the latter group. This difference is compensated by the higher frequency of wingbeat in the latter group. The calculated values of *n* resulting from Equation (4) lead in such comparisons to the need for asynchronous flight muscle in order to achieve the frequencies required. This consequence was noted in the author's earlier study (Deakin 1970).
